# 2025 Acknowledgment of *Microbiology Spectrum Ad Hoc* Reviewers

**DOI:** 10.1128/spectrum.03340-25

**Published:** 2025-12-02

**Authors:** Christina A. Cuomo

**Affiliations:** 1 Department of Molecular Microbiology and Immunology, Brown University 6752, Providence, Rhode Island, USA

## EDITORIAL

The editors of *Microbiology Spectrum* sincerely thank all the reviewers who contributed to the peer review process during the period of November 2024 through September 2025. We are grateful for the contributions of over 3,200 reviewers who have contributed their time, effort, and expertise to the journal. We would like to specifically recognize the following *ad hoc* reviewers who have reviewed three or more articles submitted to *Microbiology Spectrum*. Your dedication to the journal and its success is commendable and greatly appreciated.

Nena Abdul

Bruno L. Abbadi

Ghulam Abbas

Firouz Abbasian

April Abbott

Parveez Ahamed Abdul Azees

Neena A. Abdul Hameed

Sahar M. Abduladheem

Asra'A A. Abdul-Jalil

Masahiro Abe

Ahmed A. Abouelkhair

Walid S. Abu Rayyan

Basel H. Abuaita

Mai A. Abusalah

Debarun Acharya

Chanelle Acheamfour

La'Tonzia L. Adams

Walter Adams

Kamoru A. Adedokun

Innocent Afeke

Muhammad Afzal

Genesis Julyus T. Agcaoili

Ritesh K. Aggarwal

Stephen D. Ahator

Siddiq Akbar

Suleiman Al-Obeid

Hussein M. Al-Bayati

Oluwaseun T. Aladeboyeje

Ali Al-Ahmad

Md Tauqeer Alam

Laith K. Al-Ani

Ilyas Alav

Katie J. Aldred

Rosa Alduina

David C. Alexander

Halah H. Al-Haideri

Abdallah S. Alhaj Sulaiman

Hafidha S. Al-Hattali

Inas M. Alhudiri

Safdar Ali

Ban Al-Joubori

Mohammadreza Allahyartorkaman

Marwan Y. Al-Maqtoofi

Mushtak T. Al-Ouqaili

Mona M. Al-Shamiri

Amjed Alsultan

Hassan M. Al-Tameemi

Samuel Alvarez-Arguedas

Nana Ama Amissah

Muhammad Nabeel Amjad

Anand Anbarasu

Laura M. Andrade De Oliveira

Andres Andrade Dominguez

Medini K. Annavajhala

Winston E. Anthony

Francescopaolo Antonucci

Safaa Aqillouch

Andres Arias

Chelsie E. Armbruster

Alix Augusto Armero Villanueva

Derek T. Armstrong

Djandan Tadum Arthur Vithran

Aman Ashar

Usama Ashraf

Shahid S. Attar

Michael Aye

Shawn Babiuk

Steven F. Baker

Tanjore S. Balganesh

Louise M. Ball

Sohan R. Bangera

Bridget M. Barker

Rodolphe Barrangou

Sreekanth Reddy Basireddy

Anamika Battu

Inga Bazukyan

Wakinyan Benhamou

Fabian Berger

Richard Bernstein

Somanon Bhattacharya

Souvik Bhattacharyya

Terry W. Bilverstone

Michael Blazanin

Karin Blomqvist

Andrew J. Borchert

Michael E. Bose

Cristian Botta

Lori Bourassa

Timmie A. Britton

Titik Budiati

Michal Bukowski

Sandeepta Burgula

Fatih Buyuk

Andy Caballero Méndez

Feng Cai

José A. Calera

Juan J. Calix

Danielle E. Campbell

Yangchun Cao

Yves Carlier

Amanda C. Carroll

Santiago Castillo-Ramírez

Kaan Çeylan

Dipshikha Chakravortty

Robin R. Chamberland

Clarence W. Chan

Nora M. Chapman

Christos R. Charakas

Jayeshbhai M. Chaudhari

Changbin Chen

Qu Chen

Ying-Tsong Chen

Zhongyi Cheng

Stephen W. Chensue

Michael B. Cheung

Hosoon Choi

Saurav R. Choudhury

Rounak Chourasia

Sebastian Cifuentes

Sultan Çivi

Andrew E. Clark

Joseph M. Colacino

Shannon R. Coleman

Jerome Collemare

James Collins

Doug Cossar

Shaun T. Cross

Jie Cui

Junru Cui

Charles J. Czuprynski

Daniel M. Czyz

Flavio G. Da Fonseca

Hanchuan Dai

Ajai A. Dandekar

Lander De Coninck

Boudewijn L. De Jonge

Marija De La Marnierre

Ricardo De Souza Cardoso

Sushanta Deb

Kirk W. Deitsch

Jiaoyu Deng

Xuming Deng

Zhongliang Deng

Francisco Diez-Gonzalez

Joseph P. Dillard

Marcia Dinis

Yohei Doi

Robert G. Donald

Verónica Donato

Jakob Douan

Jingjie Du

Edward G. Dudley

Rebekah E. Dumm

Allison R. Eberly

Natalia P. Echeverría

Rubayet Elahi

Eman A. Elmansoury

Ahmed E. Elolimy

Lengsea Eng

Dean D. Erdman

German Esparza

Eduardo Espeso

Ana V. Espinel-Ingroff

Ganzhu Feng

Valentina A. Feodorova

Daniel Fernández-Soto

Paul D. Fey

María E. Flores

Francesca Florini

Bärbel U. Foesel

John A. Frank

Andrew J. Fratoni

Jonathan G. Frye

Andrei V. Gannesen

Michael G. Ganzle

Ulises Garza-Ramos

David C. Gaston

Alexandre Gouzy

Alex D. Greenwood

Charles Grose

François Guerin

Longjun Guo

Manoj Gurung

Carmen Guzmán-Bracho

Dongsheng Han

Guan-Zhu Han

Mostafa Hanafy

Nancy D. Hanson

Sohei Harada

Christopher Harmer

Lucas P. Henry

Karim Honein

Xiaoyu Hu

Lusheng Huang

James C. Hurley

Sho Iketani

Rustem A. Ilyasov

Vida R. Irani

William R. Jacobs

Leonie J. Jahn

Jichan Jang

Xinmiao Jia

Shibo Jiang

Xiaoyuan Jiang

Xingkun Jin

Tian-Zhong Jing

Jojy John

Natacha M. Juste-Poinapen

Sreenath K.

Hiroshi Kaneko

David Kaplan

Shahbaz M. Khan

Farzaneh Khodaei

Chinmayee P. Khuntia

Tomoya Kitamura

Michael Klemba

Milena Kordalewska

Michelle L. Korir

Ashish Kothari

Colleen S. Kraft

Jens Kreth

Henrike Krüger-Haker

Damian J. Krysan

Anand Kumar

Ling Ning Lam

Pierre Le Bars

Kenichi Lee

Vincent T. Lee

Patricia M. Lenhart-Pendergrass

Ingrid M. Leon

Xian-Zhi Li

Yiyuan Li

Zhanjun Li

Huimin Liu

Jianwei Liu

Yinhua Lu

Keri A. Lydon

Kevin R. Macaluso

Caroline Martini

Mathias Martins

Robert Matovu

Peter T. McKenney

Mehdi R. Mehdi Roshdi Maleki

Laura A. Mike

Adriana Miranda De Oliveira

Laxmi Mishra

Brian Mochon

Heungyun Moon

Robert B. Moreland

Luis Mota-Bravo

W. S. Moye-Rowley

Kai Mu

David R. Murdoch

Kanagavel Murugesan

Raghavendra Nagampalli

Tahereh Navidifar

Jeniel E. Nett

Peter E. Nielsen

Guoqing Niu

Lianet Noda

Dennis Nurjadi

Anthony I. Okoh

Macy G. Olson-Wood

Erika P. Orner

Kenji Ota

Thomas D. Otto

Nana Owusu-Boaitey

Erdal Özbek

Ryan M. Pace

Romina Papa-Ezdra

Hee-Soo Park

Sally R. Partridge

Matthew A. Pettengill

Brunella Posteraro

Mimi Precit

Nicole E. Putnam

Jianjun Qiao

Shangshang Qin

Joshua P. Ramsay

Jason W. Rosch

Philip J. Rosenthal

Hannah M. Rowe

Rajib Saha

Özlem Şahan Yapıcıer

Mustafa K. Şahin

Susannah J. Salter

Maurizio Sanguinetti

Patrick M. Schlievert

Bryan H. Schmitt

John L. Schmitz

Audrey N. Schuetz

Laura R. Serbus

William M. Shafer

Tariq Shah

Nana Shao

Pengfei She

Jessica R. Sheldon

Kileen L. Shier

Thomas M. Shinnick

Jerry Simecka

Ravindra P. Singh

Swadha Singh

Bahareh Sorouri

Bram Spruijtenburg

Shiranee Sriskandan

Zhongke Sun

Sarah Sundius

Milan Surjit

Sattar Taheri-Araghi

Yuki Takamatsu

Katarzyna Talaga-Ćwiertnia

Demeng Tan

Jiaxing Tan

Chao Tang

Claire S. Taylor

Benno H. Ter Kuile

Olle Terenius

Abraham Terna Lan

Michael G. Thomas

René Uebe

Shalini Upadhyay

Norman Van Rhijn

Hualong Wang

Jianxin Wang

Robert Y. Wang

Shaohua Wang

Shiwei Wang

Siran Wang

Ya Wang

Zhangxun Wang

Matthew Wargo

Nils Wetzstein

A. Christian Whelen

Dona Wijetunge

Ryan J. Williams

Dawn P. Wooley

Tong Xu

Zeling Xu

Keisuke Yaku

Bo Yan

Shangxin Yang

Tingting Yang

Cong Ye

Qing Ye

Ting-Kuang Yeh

Fangyou Yu

Yao Yu

Yunsong Yu

Chunfu Zheng

Jian Zhou

Xianfeng Zhou

Kui Zhu

Zixiang Zhu

Azna Zuberi

